# *Clitopilus lampangensis* (Agaricales, Entolomataceae), a new species from northern Thailand

**DOI:** 10.3897/mycokeys.58.36307

**Published:** 2019-09-20

**Authors:** Jaturong Kumla, Nakarin Suwannarach, Witchaphart Sungpalee, Kriangsak Sri-Ngernyuang, Saisamorn Lumyong

**Affiliations:** 1 Department of Biology, Faculty of Science, Chiang Mai University, Chiang Mai 50200, Thailand; 2 Center of Excellence in Microbial Diversity and Sustainable Utilization, Chiang Mai University, Chiang Mai 50200, Thailand; 3 Faculty of Agricultural Production, Maejo University, Chiang Mai, 50290, Thailand; 4 Faculty of Architecture and Environmental Design, Maejo University, Chiang Mai, 50290, Thailand; 5 Academy of Science, The Royal Society of Thailand, Bangkok 10300, Thailand

**Keywords:** Agaricomycetes, gill mushroom, morphology, phylogeny, tropics

## Abstract

A new species of agaricomycetes, *Clitopilus lampangensis*, is described based on collections from northern Thailand. This species was distinguished from previously described *Clitopilus* species by its pale yellow to grayish yellow pileus with the presence of wider caulocystidia. Molecular phylogenetic analyses, based on the data of the internal transcribed spacers (ITS) and the large subunit (LSU) of the nuclear ribosomal DNA, and the second largest subunit of RNA polymerase II (*rbp2*) genes, also support the finding that *C. lampangensis* is distinct from other species within the genus *Clitopilus*. A full description, color photographs, illustrations and a phylogenetic tree showing the position of *C. lampangensis* are provided.

## Introduction

The genus *Clitopilus* was proposed by Kummer (1987) with *C. prunulus* (Scop.) P. Kummer as the type species. It belongs to the family Entolomataceae of the order Agaricales. This genus is saprotrophic and is widely distributed, especially in northern temperate areas (Singer 1986; Baroni and Watling 1999; Moncalvo et al. 2002; Kirk et al. 2008; Hartley et al. 2009; Crous et al. 2012; Raj and Manimohan 2018). *Clitopilus* is characterized by basidiocarps that are clitocyboid, omphalinoid or pleurotoid, mostly whitish or occasionally grayish or brownish in color, with pink or pinkish brown spore prints, ellipsoid basidiospores with longitudinal ridges that appear angular in a polar view, and hyphae lack clamp connections (Singer 1986; Noordeloos 1988). There are 30 species of *Clitopilus* worldwide (Kirk et al. 2008), although there are 201 species names recorded in the Index Fungorum (http://www.indexfungorum.org/Names/Names.asp). The taxa list in the Index Fungorum includes synonyms and misidentifications, as well as some species that are not well documented. Formerly, the genus *Clitopilus* included *Rhodocybe* (Moncalvo et al. 2002; Co-David et al. 2009; Vizzini et al. 2011a). However, molecular phylogenetic analyses have provided powerful tools for the identification of *Clitopilus*, leading to the separation of *Clitopilus* from *Rhodocybe* as well as the related genera (*Clitocella* and *Clitopilopsis*) (Cooper 2014; Kluting et al. 2014; Raj and Manimohan 2018).

Only six species, *Clitopilus apalus* (Berk. & Br.) Petch, *C. crispus* Pat. *C. doimaesalongensis* Jatuwong, Karun. & K.D. Hyde, *C. chalybescens* T.J. Baroni & Desjardin, *C. peri* (Berk. & Br.) Petch and *C. prunulus*, have been reported in Thailand (Baroni et al. 2001; Chandrasrikul et al. 2011; Kluting et al. 2014; Jatuwong et al. 2017). During an investigation of macrofungi in northern Thailand, we found a population of *Clitopilus* which we describe here as a new species based on the morphological and molecular characteristics. To confirm its taxonomic status, the phylogenetic relationship of the new species was determined by the ITS and LSU of the rDNA, and the rbp2 genes.

## Materials and methods

### Sample collection

Basidiocarps were collected in Mae Moh District, Lampang Province, northern Thailand in 2018. Basidiocarps were wrapped in aluminum foil and kept in plastic specimen boxes to be transported to the laboratory. Notes on the macromorphological features and photographs were obtained within 24 h of collection. The specimens were dried at 40–45 °C and deposited at the Herbarium of the Sustainable Development of Biological Resources Laboratory, Faculty of Science, Chiang Mai University (**SDBR-CMU**), and BIOTEC Bangkok Herbarium (**BBH**), Pathumthani, Thailand.

### Morphological studies

Macromorphological data were recorded from fresh specimens. The recording of color names and codes followed Kornerup and Wanscher (1978). Micromorphological data were recorded from dry specimens rehydrated in 95% ethanol followed by distilled water, 3% KOH or Melzer’s reagent. Anatomical features were based on at least 50 measurements of each structure as seen under a light microscope (Olympus CX51, Japan). For spore statistics, *Q* is the ratio of spore length divided by spore width and **Q** is the average *Q* of all specimens ± standard deviation.

### Molecular phylogenetic studies

Genomic DNA of dry specimens (1–10 mg) was extracted using a Genomic DNA Extraction Mini-Kit (FAVORGEN, Taiwan). The ITS region of DNA was amplified by polymerase chain reactions (PCR) using ITS4 and ITS5 primers (White et al. 1990), the LSU of rDNA gene were amplified with LROR and LRO5 primers (Vilgalys and Hester 1990), and *rbp2* gene was amplified with the bRBP2-6F and bRBP2-7.1R primers (Matheny 2005). The amplification program for these three domains was performed in separated PCR reaction and consisted of an initial denaturation at 95 °C for 5 min, followed by 35 cycles of denaturation at 95 °C for 30 s, annealing at 52 °C for 30 s (ITS), 52 °C for 45 s (LSU), and 54 °C for 1 min (*rpb2*), and extension at 72 °C for 1 min on a peqSTAR thermal cycler (PEQLAB Ltd., UK). PCR products were checked on 1 % agarose gels stained with ethidium bromide under UV light. PCR products were purified using a PCR clean up Gel Extraction NucleoSpin Gel and PCR Clean-up Kit (Macherey-Nagel, Germany) following the manufacturer’s protocol. The purified PCR products were directly sequenced. Sequencing reactions were performed and the sequences were automatically determined in the genetic analyzer at 1^st^ Base company (Kembangan, Malaysia) using the PCR primers mentioned above. Sequences were used to query GenBank via BLAST (http://blast.ddbj.nig.ac.jp/top-e.html).

For phylogenetic analyses, the sequences from this study, previous studies and the GenBank database were used and provided in Table 1. The multiple sequence alignment was carried out using MUSCLE (Edgar 2004), and the combined ITS and LSU alignment, and *rpb2* alignment were deposited in TreeBASE under the study ID 24373 and 24374, respectively. Phylogenetic trees were constructed using maximum likelihood (ML) and Bayesian inference (BI) algorithms, implemented by RAxML v7.0.3 (Stamatakis 2006) and MrBayes v3.2.6 (Ronquist et al. 2012), respectively. *Rhodocybe griseoaurantia* and *R. pallidogrisea* were used as outgroup. The best-fit substitution model for BI and ML analyses were estimated by jModeltest 2.1.10 (Darriba et al. 2012) using Akaike information criterion (AIC). For ML analysis, the bootstrap (BS) replicates were set as 1000 and used to test phylogeny (Felsenstein 1985). Clades with bootstrap values (BS) of ≥ 70% were considered significantly supported (Hillis and Bull 1993). For the BI analysis, the Markov chains were run for one million generations, with six chains and random starting trees. The chains were sampled every 100 generations. Among these, the first 2,000 trees were discarded as burn-in, while the postburn-in trees were used to construct the 50% majority-rule consensus phylogram with calculated Bayesian posterior probabilities. Bayesian posterior probabilities (PP) ≥ 0.95 were considered significant support (Alfaro et al. 2003).

**Table 1.** Sequences used for phylogenetic analysis. The newly generated sequences are in bold.

Taxa	Voucher/strain	GenBank accession number			Refernces
		ITS	LSU	*rpb2*	
*Clitopilus albidus*	CAL 1320	MF926596	MF926595	MF946579	Raj and Manimohan 2018
	CORT:26394WAT	–	KR869936	KC816906	Largent and Bergemann 2016
	M536	–	AF261287	–	Moncalvo et al. 2002
*Clitopilus austroprunulus*	MEN2009062	KC139085	–	–	Phillips and Dinis 2012
	MEN2009001	KC139084	–	–	Phillips and Dinis 2012
Clitopilus cf. argentinus	MTB480412	–	–	KC816907	Kluting et al. 2014
*Clitopilus chalybescens*	MFUCC130808	KP938184	–	–	Jatuwong et al. 2017
	MFUCC130809	KP938185	–	–	Jatuwong et al. 2017
	SDBR-CMUUP0039	**MK773645**	**MK764940**	**MK784129**	This study
*Clitopilus chrischonensis*	TOHG 1994	HM623128	HM623131	–	Vizzini et al. 2011b
*Clitopilus crispus*	GDGM29931	JQ281489	–	–	He et al. 2012
	CORT:9982	–	–	KC816910	Kluting et al. 2014
	CORT:10027	–	–	KC816911	Kluting et al. 2014
*Clitopilus cystidiatus*	26	–	GQ289147	GQ289220	Co-David et al. 2009
	TOAV130	HM623129	HM623132	–	Vizzini et al. 2011b
*Clitopilus doimaesalongensis*	MFUCC130806	KP938183	–	–	Jatuwong et al. 2017
*Clitopilus fusiformis*	SAAS1038	KY385634	–	KY385632	Wang et al. 2017
	SAAS1892	KU751777	–	KY385633	Wang et al. 2017
*Clitopilus giovanellae*	SF14368	EF413030	EF413027	–	Moreno et al. 2007
*Clitopilus hobsonii*	CBS 270.36	FJ770395	–	–	Hartley et al. 2009
	CBS 445.86	FJ770385	–	–	Hartley et al. 2009
	DLL9635	–	–	KC816913	Kluting et al. 2014
	DLL9643	–	–	KC816913	Kluting et al. 2014
*Clitopilus lampangensis*	SDBR-CMUJK 0147	**MK764933**	**MK764935**	**MK784127**	This study
	SDBR-CMUNK 0047	**MK764934**	**MK773856**	**MK784128**	This study
*Clitopilus kamaka*	KA12-0364	KR673433	–	–	Kim et al. 2015
*Clitopilus orientalis*	CAL 1616	MG345134	MG321558	MG321559	Raj and Manimohan 2018
*Clitopilus passeckeriamus*	CBS299.35	MH855682	MH867198	–	Vu et al. 2019
	P78	KY962494	KY963078	–	Unpublished
*Clitopilus paxilloides*	CORT:5809	–	–	KC816919	Kluting et al. 2014
*Clitopilus peri*	CORT:10033	–	–	KC816920	Kluting et al. 2014
	CORT:10040	–	–	KC816921	Kluting et al. 2014
	CORT:10041	–	–	KC816922	Kluting et al. 2014
*Clitopilus pinsitus*	CBS 623.70	MH859879	MH871665	–	Vu et al. 2019
*Clitopilus prunulus*	Champ-15	KX449418	–	–	Pérez-Lzquierdo et al. 2017
	CBS 227.93	FJ770408	–	–	Hartley et al. 2009
	Noordeloos 2003-09-14	KR261096	–	–	Unpublished
	COPT:7003	–	–	KC816925	Kluting et al. 2014
	TB9663	–	–	GU384648	Baroni et al. 2011
	TB8229	–	–	GU384650	Baroni et al. 2011
	COPT:REH8456	–	–	KC816923	Kluting et al. 2014
*Clitopilus reticulosporus*	DC-2010	KC885966	HM164414	HM164416	Vu et al. 2019
*Clitopilus scyphoides*	CBS 127.47	MH856181	MH867707	–	Vu et al. 2019
	CBS 400.79	FJ770401	–	–	Hartley et al. 2009
*Clitopilus subscyphoides*	CAL 1325	MF927542	MF946580	MF946581	Raj and Manimohan 2018
*Clitopilus venososulcatus*	CORT:8111	–	–	KC816930	Kluting et al. 2014
*Rhodocybe griseoaurantia*	CAL 1324	KX083571	KX83574	KX083568	Unpublished
*Rhodocybe pallidogrisea*	CORT 013944	NR154437	–	KC816968	Kluting et al. 2014

## Results

### Phylogenetic analyses

**Figure 1.**
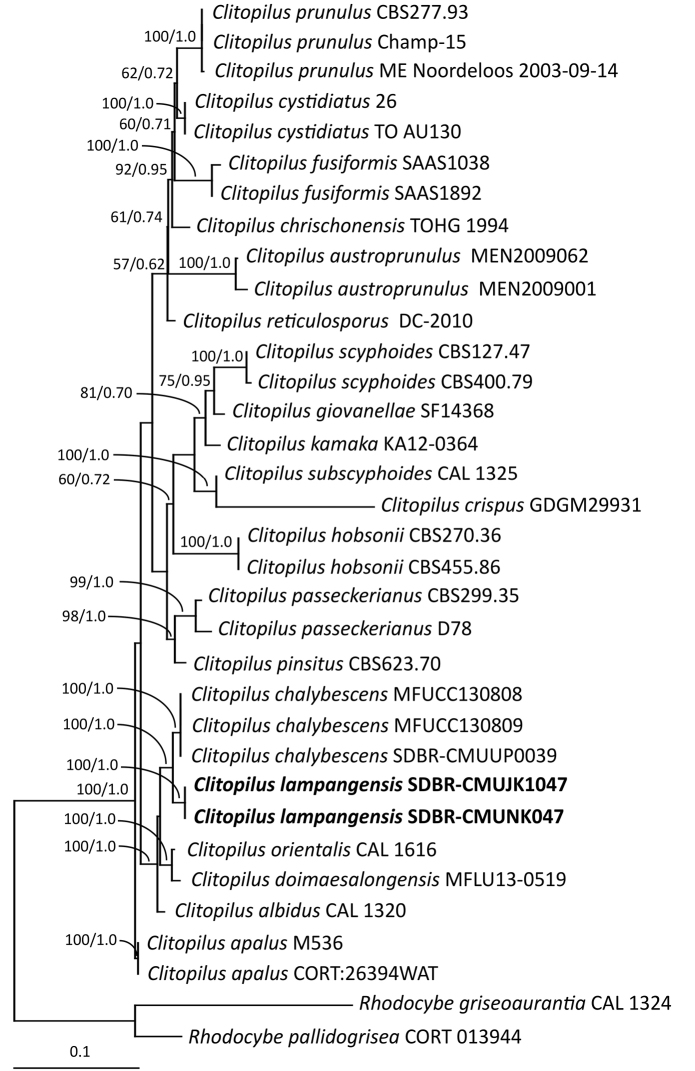
Phylogram derived from maximum likelihood analysis of the combined ITS and LSU region of nuclear rDNA of 34 sequences. *Rhodocybe griseoaurantia* and *R. pallidogrisea* were used as outgroup. The numbers above branches represent maximum likelihood bootstrap percentages (left) and Bayesian posterior probabilities (right). Only bootstrap values ≥ 50 % are shown, and the scale bar represents ten substitutions per nucleotide position. The fungal species obtained in this study are in bold.

**Figure 2.**
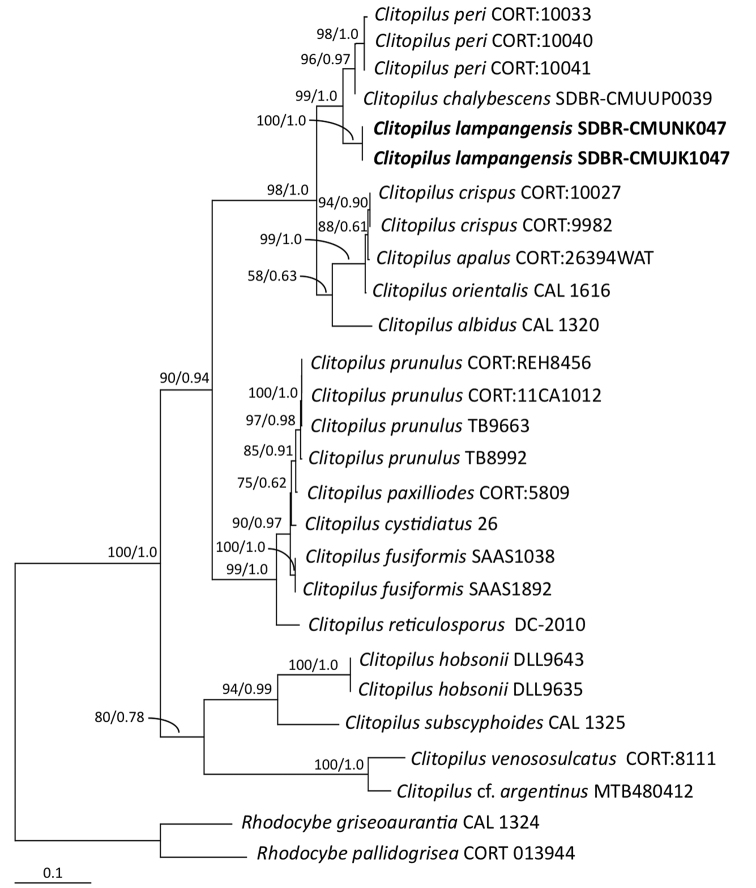
Phylogram derived from maximum likelihood analysis of *rpb2* gene of 27 sequences. *Rhodocybe griseoaurantia* and *R. pallidogrisea* were used as outgroup. The numbers above branches represent maximum likelihood bootstrap percentages (left) and Bayesian posterior probabilities (right). Only bootstrap values ≥ 50 % are shown, and the scale bar represents ten substitutions per nucleotide position. The fungal species obtained in this study are in bold.

The topology of each single-gene of ITS and LSU, and the combined ITS and LSU phylograms were found to be similar. However, differences were observed in the topology of the *rbp2* gene. Therefore, we present only the combined ITS and LSU gene phylogram (Fig. 1), and the single *rbp2* gene phylogram (Fig. 2). The combined ITS and LSU sequence dataset consisted of 34 taxa and were comprised of 1774 characters including gaps (ITS: 1–779, LSU: 780–1774). The sequence dataset of *rbp2* consisted of 27 taxa and the aligned dataset was comprised of 620 characters that included gaps. The GTR model with gamma rate heterogeneity and invariant sites (GTR+G+I) was the best-fit model used for both ML and BI analyses that were selected by AIC. The average standard deviation of the split frequencies fell to 0.011364 and 0.009837 in the BI analysis of the combined ITS and LSU, and *rbp2* sequences, respectively after one million generations. This was observed after the 50% majority consensus phylogram was constructed. The ML analysis of the combined ITS and LSU sequences was based on the parameters estimated under the GTR+I+G model, and the proportion of the invariable sites and the gamma shape parameters were 0.0250 and 0.9320, respectively. Additionally, the tree with log likelihood (-8211.7515) was built after 1000 bootstrapping replications. In the ML analysis of the *rbp2* sequence that was based on the GTR+I+G model, the proportion of the invariable sites and the gamma shape parameters were 0.5400 and 1.7960, respectively, while the tree with log likelihood (-3640.1616) was built after 1000 bootstrapping replications.

Both the combined ITS and LSU, and the *rbp2* phylograms indicated that the sequences were of a new species, *C. lampangensis*, that had formed a monophyletic clade with high BS (100 %) and PP (1.0) support (Figs 1, 2). A combined ITS and LSU phylogram revealed that the new species was a sister taxon to *C. chalybescens*. In addition, the *rbp2* phylogram indicated that the new species was a sister taxon to *C. chalybescens* and *C. peri*.

### Taxonomy

#### Clitopilus lampangensis

Taxon classificationFungiAgaricalesEntolomataceae

J. Kumla, N. Suwannarach & S. Lumyong sp. nov.

CA17D2DA-0571-547D-B81E-EF13D851DEAF

830890

Fig. 3

##### Diagnosis.

Distinguished from other *Clitopilus* species by its pale yellow to grayish yellow pileus with the presence of caulocystidia, and from *C. chalybescens* by its wider caulocystidia, longer basidiospores, and lack of grayish blue color change on the pileus and stipe when bruised.

##### Etymology.

‘*lampangensis*’, referring to Lampang Province, where the holotype was found.

##### Holotype.

THAILAND, Lampang Province, Mae Moh District, (18°24'21"N, 99°42'26"E, elevation 380 m), on ground in a tropical deciduous forest, May, 2018, J. Kumla & N. Suwannarach, SDBR-CMUJK 0147 and BBH 43590 (isotype).

##### Gene sequence (from holotype).

MK764933 (ITS), MK764935 (LSU) and MK784127 (*rbp2*).

Basidiocarps small, clitocyboid. Pileus 35–50 mm diam., initially convex or somewhat plano-convex with or without a central depression, becoming deeply umbilicate with age; surface pale yellow (4A3) to greyish yellow (4B5), somewhat velutinous, finely pruinose all over; margin incurved to slightly inrolled, entire or slightly wavy. Lamellae subdecurrent to decurrent, white (1A1), crowded, up to 2.5 mm wide, with lamellulae of 1–3 lengths; edge entire or slightly wavy, concolorous with the sides. Stipe 20–25 × 5–8 mm, central, solid; surface white (1A1) to yellowish white (4A2), finely pruinose all over, densely so towards the apex; base with white cottony mycelium. Odor strong farinaceous. A pale pinkish spore print.

Basidiospores 7.0–9.0 × 3.0–5.0 μm, *Q* = 1.40–2.33, **Q** = 1.82 ± 0.27, ellipsoid in polar view, amygdaliform to limoniform in side view, with 6–8 prominent longitudinal ridges, colorless, thin-walled. Basidia 17.0–25.0 × 4.0–8.0 μm, clavate, colorless, thin-walled, 2- and 4-spored; sterigmata up to 4 μm long. Lamella-edge fertile. Pleurocystidia and cheilocystidia absent. Lamellar trama subregular; hyphae 2.5–4.0 μm wide, hyaline, thin-walled. Pileus trama compact, hyaline, cylindrical hyphae 5–10 μm wide. Pileipellis a cutis of loosely interwoven hyphae; 3–5 μm wide, hyaline, thin-walled, and terminal cells; subcylindric or narrowly clavate, 4–8 μm wide. Stipitipellis at stipe apex a layer of repent, hyaline, cylindrical hyphae 4–8 μm wide, thin-walled. Caulocystidia 25.5–42.5 × 8.0–15.0 μm, single or clustered, erect or repent, varying in shape from cylindrical to clavate, hyaline, slightly thick-walled. Clamp connections absent in all tissues.

**Figure 3.**
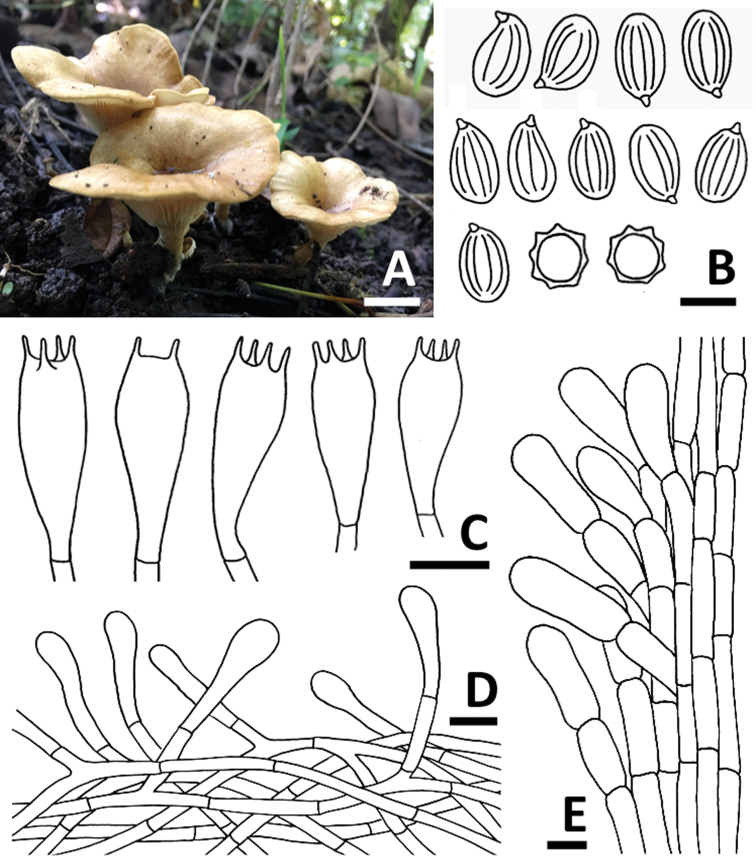
*Clitopilus lampangensis* SDBR-CMUJK 0147 (holotype). **A** Basidiocarps **B** Basidiospores **C** Basidia **D** Pileipellis **E** Caulocystidia. Scale bars: 10 mm (**A**), 5 μm (**B**), 10 μm (**C–E**).

##### Ecology and distribution.

Fruiting solitary or gregarious on soil in a tropical deciduous forest. Known only from northern Thailand

##### Specimens examined.

THAILAND, Lampang Province, Mae Moh District, (18°24'20"N, 99°42'3"E, elevation 375 m), on ground in a tropical deciduous forest, May, 2018, N. Suwannarach & J. Kumla, SDBR-CMUNK 0047, GenBank sequence MK764934 (ITS), MK773856 (LSU) and MK784128 (*rbp2*).

## Discussion

The present study has identified a new species of *Clitopilus* acquired from northern Thailand based on both morphological characteristics and phylogenetic analyses. *Clitopilus lampangensis* is characterized by its clitocyboid, pale yellow to grayish yellow basidiocarps, pinkish spore-print, ellipsoid basidiospores with longitudinal ridges and hyphae lacking clamp connections. Thus, these morphological characteristics support its placement into the genus *Clitopilus* (Singer 1986; Noordeloos 1988). Based on the morphology, the pale yellow to grayish yellow pileus of *C. lampangensis* distinguishes it from the white and grayish pileus of *Clitopilus* species, with the exceptions of *C. catalonicus*, *C. djellouliae*, *C. fasciculatus*, *C. gallaecicus*, *C. giovanellae*, *C. incrustatus*, *C. luteocinnamomeus* and *C. prunulus*, (Kummer 1871; Singer 1942; Noordeloos 1984; Baroni and Halling 2000; Moreno et al. 2007; Ovrebo and Baroni 2007; Vila et al. 2008; Contu et al. 2011; Desjardin et al. 2015). The characteristics of the basidiocarps and size of the basidia, caulocystidia and basidiospores of *C. lampangensis* were compared with related *Clitopilus* species (Table 2). The presence of caulocystidia in *C. lampangensis* clearly distinguishes it from these related species. Moreover, the pileus of *C. lampangensis* (35–50 mm in diameter) are larger than *C. djellouliae* (6–18 mm in diameter; Contu et al. (2011)), *C. giovanellae* (5–15 mm in diameter; Singer (1942) and Moreno et al. (2007)) and *C. catalonicus* (up to 15 mm in diameter; Vila et al. (2008)). Prior to this study, *C. apalus*, *C. crispus*, *C. doimaesalongensis*, *C. chalybescens*, *C. peri* and *C. prunulus* had been found in Thailand (Baroni et al. 2001; Chandrasrikul et al. 2011; Kluting et al. 2014; Jatuwong et al. 2017). However, *C. apalus*, *C. crispus*, *C. peri* and *C. doimaesalongensis* differ from *C. lampangensis* by their white to chalk-white pileus and a lack of caulocystidia (Pegler 1986; Yang 2000; Jatuwong et al. 2017). The larger basidia and basidiospores, and the absence of caulocystidia in *C. prunulus* clearly differentiate it from *C. lampangensis* (Kummer 1871; Desjardin et al. 2015) (Table 2). Both *C. lampangensis* and *C. chalybescens* have caulocystidia (Baroni et al. 2001; Jatuwong et al. 2017). However, the width of the caulocystidia and the length of the basidiospores of *C. chalybescens* are narrower and shorter than in *C. lampangensis* (Table 2) (Baroni et al. 2001; Jatuwong et al. 2017).

**Table 2.** Comparison of *Clitopilus lampangensis* with the closely related species.

Taxa	Origin	Pileus	Basidia	Caulocystidia	Basidiospores
*C. lampangensis* ^a^	Thailand	35–50 mm in diameter, pale yellow to greyish yellow	17.0–25.0 × 4.0–8.0 μm, 2–4 streigmata	25.5–42.5 × 8.0–15.0 μm	Ellipsoid, 7.0–9.0 × 3.0–5.0 μm, 6–8 longitudinal ridges
*C. chalybescens* ^b, c^	Thailand	15–90 mm in diameter, white, yellowish white to greyish blue	15.0–21.0 × 5.1–8.0 μm, 4 streigmata	16.0–32.0 × 5.0–7.0 μm	Ellipsoid, 5.3–7.5 × 3.6–5.0 μm, 8–10 longitudinal ridges
*C. peri* ^d,e^	India, Sri Lanka, Thailand	8–22 mm in diameter, white	16.0–18.0 × 5.0–7.0 μm, 4 streigmata	Absent	Ellipsoid, 6.7–8.5 × 3.0–4.0 μm, 6–9 longitudinal ridges
*C. prunulus* ^f,g^	Netherlands, Thailand, United State	25–80 mm in diameter, white, yellowish white to grayish or yellow cream	25.0–47.0 × 7.0–12.0 μm, 4 streigmata	Absent	Ellipsoid, 9.0–14.0 × 4.5–8.0 μm, 6–8 longitudinal ridges
*C. fasciculatus* ^h^	Netherlands,	20–70 mm in diameter, pale brown	Sizes were not reported, 4 streigmata	Absent	Ellipsoid, 4.5–6.3 × 3.0–4.0 μm, 3–6 longitudinal ridges
*C. gallaecicus* ^i^	Spain	80–90 mm in diameter, creamy, ochre to ochre-brown	20.0–35.0 × 8.5–10.5 μm, 4 streigmata	Absent	Ellipsoid, 8.0–14.5 × 4.5–7.5 μm, 3–6 longitudinal ridges
*C. incrustatus* ^j^	Costa Rica, United State	80–90 mm in diameter, grayish brown	16.0–24.0 × 7.0–8.0 μm, 4 streigmata	Absent	Ellipsoid, 5.0–6.5 × 3.0–4.0 μm, 3–6 longitudinal ridges
*C. djellouliae* ^k^	France	6–18 mm in diameter, light yellowish brown	22.0–32.0 × 7.5–8.5 μm, 4 streigmata	Absent	Ellipsoid, 6.0–9.0 × 4.0–6.0 μm
*C. giovanellae* ^l,m^	Italy, Spain	5–15 mm in diameter, grayish to light brown	14.0–22.0 × 6.5–9.5 μm, 4 streigmata	Absent	Ellipsoid, 5.0–8.0 × 3.0–4.0 μm
*C. luteocinnamomeus* ^n^	Panama	15–45 mm in diameter, ochre to light cinnamon-brown	19.0–27.0 × 6.0–7.0 μm, 4 streigmata	Absent	Subglobose to ellipsoid, 4.5–6.0 × 3.5–5.0 μm
*C. catalonicus* ^o^	Panama	Up to 15mm in diameter, light yellowish brown	32.0–40.0 × 6.4–8.0 μm, 4 streigmata	Absent	Ellipsoid, 5.3–7.5 × 3.7–4.5 μm

^a^This study, ^b^Baroni et al. (2001), ^c^Jatuwong et al. (2017), ^d^Pegler (1986), ^e^Kluting et al. (2014), ^f^Kummer (1871), ^g^Desjardin et al. (2015), ^h^Noordeloos (1984), ^i^Blanco-Dios (2013), ^j^Baroni and Halling (2000), ^k^Contu et al. (2011), ^l^Singer (1942), ^m^Moreno et al. (2007), ^n^Ovrebo and Baroni (2007) and ^o^Vila et al. (2008).

The phylogenetic analyses of the combined ITS and LSU, and *rpb2* sequences confirmed that *C. lampangensis* formed a monophyletic clade which clearly separated it from the other *Clitopilus* species. *Clitopilus lampangensis* forms a sister taxon to *C. chalybescens* and *C. peri*. *Clitopilus peri* differs from *C. lampangensis* by its smaller white basidiocarps (8–22 mm in diameter) and the absence of caulocystidia (Pegler 1986). Additionally, the different morphological characteristics that exist between *C. lampangensis* and *C. chalybescens* have been mentioned above.

Therefore, a combination of the morphological characteristics and the molecular analyses strongly support recognition of a new fungus species. This discovery is considered important in terms of stimulating a deeper investigation of macrofungi in Thailand, and will help researchers to better understand the distribution and ecology of *Clitopilus*.

### Key to *Clitopilus* species known from Thailand

**Table:** 

1	Pileus white to chalk-white colors	**2**
–	Pileus white or with other colors	**5**
2	Stipe ≥ 3 mm thick	**3**
–	Stipe < 3 mm thick	***C. peri***
3	Basidia < 8 μm wide	**4**
–	Basidia ≥ 8 μm wide, basidiospores 6.8–9.2 × 4.1–5.5 μm	***C. doimaesalongensis***
4	Basidia up to 25 μm, basidiospores 6–8.5 × 4.5–5.5 μm	***C. apalus***
–	Basidia up to 30 μm, basidiospores 5.5–9 × 4–6 μm	***C. cripus***
5	Pileus white to pale grayish or yellowish cream colors	**6**
–	Pileus pale yellow to greyish yellow colors, caulocystidia present, basidiospores 7.0–9.0 × 3.0–5.0 μm	***C. lampangensis***
6	Basidia ≥ 25 μm long, caulocystidia absent, basidiospores 8.0–12.0 × 4.0–6.5 μm	***C. prunulus***
–	Basidia < 25 μm long, caulocystidia present, basidiospores 5.3–7.5 × 3.6–5.0 μm	***C. chalybecens***

## Supplementary Material

XML Treatment for Clitopilus lampangensis
